# Connexin Regulation and Modulation of Neural Stem Cell Differentiation Induced by Cell‐Permeable Itaconate

**DOI:** 10.1002/jcp.70185

**Published:** 2026-05-11

**Authors:** Simona Denaro, Simona Rosa Spina, Simona D'Aprile, Anna Gervasi, Filippo Torrisi, Carmela Parenti, Agata Zappalà, Nunzio Vicario, Rosalba Parenti

**Affiliations:** ^1^ Department of Biomedical and Biotechnological Sciences University of Catania Catania Italy; ^2^ Department of Medicine and Surgery University of Enna “Kore” Enna Italy; ^3^ Department of Drug and Health Sciences University of Catania Catania Italy

**Keywords:** dimethyl itaconate, gap junction, intercellular communication, neural progenitors, subventricular zone

## Abstract

Neural stem cells (NSCs) are multipotent cells of the central nervous system (CNS) capable of self‐renewal, differentiation, and responding to and shaping the surrounding microenvironment. Their continuous crosstalk with surrounding CNS cells is a key component of their therapeutic potential, particularly in tissue repair and regeneration. Communication in the CNS relies on complementary mechanisms, including connexins (Cxs)‐based intercellular communication, to maintain homeostasis and coordinate responses to physiological and pathological stimuli. Itaconate, an endogenous shunt product of the tricarboxylic acid cycle, functions as an immunometabolite involved in inflammation and oxidative stress and has recently been implicated in neuroimmune modulation. Although itaconate influences several signalling cascades and is exchanged between cells and/or released into the extracellular milieu, its effects on Cxs expression in NSCs and whether the modulation of Cxs expression profile represents a driving factor in shaping cell fate remain unclear. Here, we investigated the effect of dimethyl itaconate, a cell‐permeable esterified itaconate derivative, on the expression profile of Cxs in NSCs and its potential to modulate NSCs fate and differentiation. We found that dimethyl itaconate modulates Cxs expression in NSCs, increasing Cx36 levels, and promotes NSCs differentiation toward a neuronal phenotype, while inhibition of Cxs‐based channels with carbenoxolone or mefloquine abolishes these dimethyl itaconate‐induced effects. Collectively, these findings highlight a regulatory role for cell‐permeable itaconate and contribute to the understanding of intercellular communication in the CNS microenvironment, providing insights into potential therapeutic strategies for CNS repair and regeneration.

## Graphical abstract

The immunometabolite itaconate modulates the connexin profile of neural stem cells, and it upregulates Cx36‐based channels, promoting the differentiation toward a neuronal phenotype. These effects were abolished by connexin blockade, supporting a role for itaconate in the neuroimmune axis in inflammatory and neurodegenerative disorders.

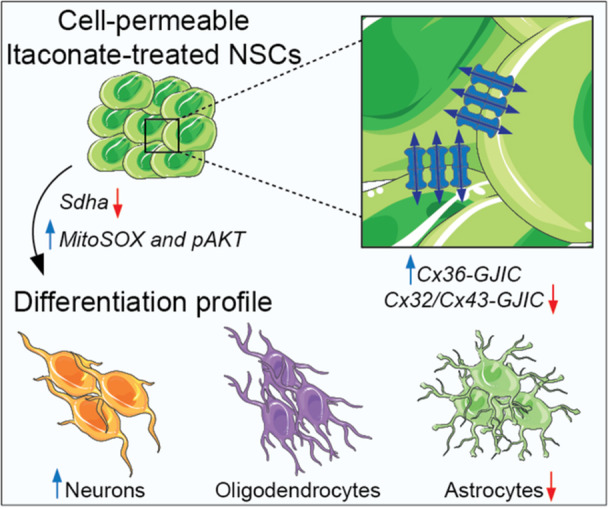

## 1 Introduction

Neural stem cells (NSCs) are multipotent cells in the central nervous system (CNS) defined by their intrinsic capacity to self‐renew and give rise to neurons, astrocytes, and oligodendrocytes (Vescovi and Snyder 1999). Their functions are not restricted to the embryonic phase, as NSCs retain the ability to stimulate endogenous or exogenous repair mechanisms and to sense and respond to stimuli from the surrounding microenvironment in the adult CNS (Nunes et al. 2003; Thored et al. 2006). NSCs localise in at least two canonical niches: the subventricular zone (SVZ) and the subgranular zone (SGZ). Additional non‐canonical or less active niches that may persist in adulthood include the hypothalamus, striatum, spinal cord, and cerebellum (Batailler et al. 2016; Finkel et al. 2021; Marichal et al. 2017). In both the SVZ and the SGZ, NSCs are characterised by long‐term self‐renewal, proliferative potential, and the ability to differentiate (Andreotti et al. 2019). These niches also comprise microglia, endothelial cells of blood vessels and their basal lamina, perivascular cells, and axon terminals (Lim et al. 2000). Notably, microglial processes are closely associated with blood vessels and astrocytes, influencing NSCs' survival and self‐renewal (Solano Fonseca et al. 2016). NSCs are active players in the crosstalk with microglia and infiltrating monocyte‐derived macrophages, responding to various inducers, sensors, transducers, and effectors of neuroinflammation, including small metabolites (Peruzzotti‐Jametti et al. 2018). Although cell replacement and pro‐regenerative effects of NSC‐derived factors are considered fundamental to NSC‐based approaches, continuous and dynamic crosstalk with the CNS microenvironment and resident cells is also pivotal to their therapeutic potential (Denaro et al. 2022; Yamada et al. 2024).

Mechanisms underlying intercellular communication include neurotransmitters, paracrine factors, extracellular vesicles, tunnelling nanotubes, ion channels, and membrane transporters (Berumen Sánchez et al. 2021; Vicario et al. 2020; Zhou et al. 2024). Interaction between neighbouring cells also relies on gap junction intercellular communication (GJIC). Gap junctions (GJs) consist of two hemichannels (HCs) that connect the intracellular fluids of two adjacent cells. Each HC is composed of six connexin (Cx) subunits, which are a family of transmembrane proteins encoded by 21 different genes, with specific features characterising their structure, half‐life, and functions (Denaro et al. 2025). Importantly, HCs not only dock with each other but can also release factors into the extracellular milieu, thus working as membrane pores with either autocrine or paracrine functions. Given that different Cxs may form a single HC or that GJs may consist of HCs with different Cxs, homomeric or heteromeric HCs and homotypic or heterotypic GJs have been described (Vicario and Parenti 2022).

In the adult CNS, neurons typically express Cxs‐based GJs at the level of electrical synapses or HCs activated to sustain purinergic signalling. The most abundant neuronal Cx is the Cx36, which is associated with neuronal firing and communication and is considered a marker of functional neurons (Siu et al. 2016). Oligodendrocytes are characterised by Cx32 expression, which has been implicated in the process of myelination, maturation, and communication between myelin layers (Nualart‐Marti et al. 2013). Astrocytes typically express high levels of GJs‐forming proteins, particularly Cx30 and Cx43, and their functions become critical when astrocytes interact with CNS resident immune cells (Denaro et al. 2024; Denaro et al. 2024; Vicario et al. 2017).

SVZ‐derived NSCs express high levels of Cxs, which are functionally important and required to form cellular networks that synchronise calcium ion activity and stimulate cell proliferation (Malmersjö et al. 2013; Ravella et al. 2015). Evidence suggests that this intercellular network influences NSCs' survival and differentiation (Genet et al. 2023). While homeostasis and small‐world networks within stem cell niches contribute to their maintenance, these niches are also exposed to stimuli from other resident CNS cell populations (Gabel et al. 2016). Released extracellular mediators, such as growth factors, cytokines, and signalling molecules, condition the CNS microenvironment, exerting context‐dependent supportive or detrimental effects on NSCs (Kizil et al. 2015). Increasing evidence indicates that cellular metabolism and redox signalling play central roles in regulating stem cell fate and niche dynamics.

Itaconate, an endogenous shunt product of the tricarboxylic acid cycle, and its derivatives have been related to critical immunomodulatory and metabolic functions, including regulation of immune cells, inflammation, and oxidative stress (Hooftman et al. 2020; Kuo et al. 2020). Itaconate is considered a short‐lived and signalling metabolite synthesised via the decarboxylation of cis‐aconitate within mitochondria in response to immune‐responsive gene 1 (IRG1) induction (Ohm et al. 2024; Watermann et al. 2024). Although itaconate is barely detectable or absent under homeostatic conditions, its intracellular concentration can reach 500−1000 μM in LPS‐ or LPS/IFN‐γ‐primed monocytes/macrophages (Chen et al. 2022). In particular, it acts as an inhibitor for succinate dehydrogenase (SDH) to exert its modulatory functions on metabolism, leading to succinate accumulation and redox balance alterations (Murphy and O'Neill 2018). Such accumulation has been associated with increased production of reactive oxygen species (ROS), in turn acting on Nuclear Factor Erythroid 2‐related factor 2 (NRF2) and the Akt signalling pathways (Hu et al. 2024; Murphy and O'Neill 2018). These redox‐dependent mechanisms align with the broader concept that metabolic reprogramming and redox signalling modulate NSCs differentiation, acting together with NRF2 to balance self‐renewal and lineage commitment (Dai et al. 2020). Moreover, itaconate has been associated with the activation of NRF2, mediating stress response mechanisms via alkylation of cysteine residues on Keap1 in both mouse and human macrophages (Mills et al. 2018). However, inhibition of SDH alone does not fully account for the broad immunoregulatory effects observed with the esterified cell‐permeable derivative dimethyl itaconate (DMI). In this context, activation of the NRF2 pathway in response to electrophilic stress has also been reported to trigger a secondary, independent, transcriptional programme following Toll‐like receptor stimulation via ATF3‐mediated inhibition of IkappaB‐zeta (IκBζ) (Bambouskova et al. 2018). Additional functions of itaconate have been reported, including the suppression of bacterial growth in macrophages via activation of the lysosomal biogenesis factor transcription factor EB (TFEB), resulting in autophagy‐mediated pathogen degradation (Schuster et al. 2022). Experimental evidence has shown opposite effects of itaconate and its esterified derivatives on modulation of the Akt signalling pathway in immune cells, although a significant upregulation of pAkt levels upon 4‐octyl itaconate treatment has been observed in an in vitro model of oxidative stress (Hu et al. 2024; Sohail et al. 2022).

Given that ROS and Akt signalling influence Cxs‐based channels and NSCs fate (Le Belle et al. 2011; Park et al. 2007), it is plausible that itaconate‐mediated metabolic reprogramming could influence intercellular communication within the NSCs' niches. Furthermore, as itaconate can be released from activated immune cells and potentially diffuse or be transported through pore‐forming channels, crosstalk between immune‐derived metabolites and NSCs may occur within the CNS microenvironment. Despite growing interest in itaconate's immunometabolic functions, its effects on Cxs expression patterns and NSCs differentiation potential remain unexplored. Here, we hypothesised that the fate and differentiation of SVZ‐derived NSCs may be influenced by itaconate released by immune cells, and we investigated the effect of its cell‐permeable derivative, DMI, on Cxs expression and NSCs differentiation. We also focused on the observed increased expression levels of Cx36, assessing its role in intercellular communication and modulating its channel‐forming function.

## 2 Materials and Methods

### 2.1 NSCs Derivation, Culture, and Differentiation

NSCs were derived from the SVZ of 8−12‐week‐old male C57BL/6 mice (Vescovi and Snyder 1999). Brains from 2 to 4 animals were isolated, washed in EBSS (Cat. no. 24010‐043, Gibco), coronal 3 mm‐thick sections were collected, and SVZs were isolated and pooled. Dissected tissues were transferred into a digestion medium composed of 1 mg/mL papain (Cat. no. LS003126, Worthington), 0.2 mg/mL ethylenediaminetetraacetic acid (EDTA, Cat. no. E9884, Sigma‐Aldrich), and 0.2 mg/mL L‐cysteine (Cat. no. J63745, Alfa‐Aesar) in EBSS and incubated for 45 min at 37°C. The suspension was then centrifuged at 200*g* for 12 min, and the pellet was mechanically triturated in 2 mL of EBSS. After an additional centrifugation at 200*g* for 12 min, the cell pellets were resuspended and seeded in complete growth medium composed of 2 μg/mL heparin (Cat. no. H3393, Sigma‐Aldrich), 20 ng/mL EGF (Cat. no. AF‐100‐15, PeproTech), and 10 ng/mL bFGF (Cat. no. 100‐18B, PeproTech) in NeuroCult basal medium (Cat. no. 05700, Stem Cell Technologies) plus mouse NeuroCult proliferation supplements (Cat. no. 05701, Stem Cell Technologies). After 4−6 days, neurospheres were collected and enzymatically digested with Accutase (Cat. no. A11105‐01, Gibco) at 37°C for 10 min. The number of viable cells was determined by trypan blue exclusion, and cells were replated at a clonal density of 10,000 cell/cm^2^.

The rate of cell growth (R.C.G.) was calculated by counting the number of viable cells by trypan blue exclusion and dividing it by the number of plated cells. This value was normalised by dividing it by the number of days per passage (Torrisi et al. 2021). The linear growth curve was generated by estimating the total number of cells by multiplying the R.C.G. by the total number of cells at the previous passage (Supporting Information S1: Figure S1).

For the NSCs differentiation assay, cells were seeded on a 13 mm round coverslip precoated with Matrigel (Cat. no. 356234, Corning) at a density of 80,000 cells per coverslip and cultured in 400 μL of differentiation medium composed of 10% mouse differentiation supplement (Cat. no. 05703, Stem Cell Technologies) in NeuroCult basal medium (Cat. no. 05700, Stem Cell Technologies). Half of the medium was replaced with fresh differentiation medium after 3 days of culture. Differentiated cells were fixed after 6 days.

### 2.2 MTT‐Turnover and LDH Assays

Cells were seeded in 96‐well plates at a final density of 10,000 cells per well in 100 μL of medium supplemented with laminin (Cat. no. 11243217001, Roche, 1:100) and incubated for 24 h. The following day, cells were exposed to DMI (Cat. no. 592498, Sigma‐Aldrich) diluted in growth medium at a final concentration of 0.1, 1, 10, 100, or 1000 μM in DMSO (as specified in the figure legend) and incubated for 24 h. For DMI treatment, a 25 mM stock solution of DMI (Cat. no. 592498, Sigma‐Aldrich) was prepared in DMSO, and DMI was added to cultures in the medium at a maximum concentration of 1.0%. Control groups received an equal amount of DMSO.

3‐(4,5‐dimethylthiazol‐2‐yl)‐2,5‐diphenyltetrazolium bromide (MTT, Cat. no. M5655, Sigma‐Aldrich) was added to each well at a final concentration of 1 mg/mL, and cells were incubated for 3 h under standard culture conditions (37°C, 5% CO_2_). Then, the media were removed, and 200 μL of DMSO was added to each well. Plates were stirred on an orbital shaker for 10 min at room temperature, and the absorbance was measured using a Multiskan SkyHigh Microplate Reader spectrophotometer at 570 nm. Metabolic turnover was calculated as the optical density of each sample divided by the mean optical density of control cultures and expressed as a percentage.

The determination of extracellular lactate dehydrogenase (LDH) was performed using the above‐described plating and treatment conditions. 24 h after treatment, 20 μL of Triton X‐100 was added to the LDH positive control and the same amount of PBS to all other conditions. After 2 h, the medium from each well was collected and centrifuged at 13,000 rpm for 5 min. In a new 96‐well plate, 50 μL of PBS was mixed with 40 μL of collected medium and 10 μL of LDH reagent (Cat. no. 124101, Cell Biolabs), according to the manufacturer's instructions. The plate was incubated for 1 h at 37°C in 5% CO_2_. Relative cytotoxicity was calculated using the following formula, where optical density (OD) refers to the absorbance at 450 nm, measured using a Multiskan SkyHigh Microplate Reader:

$$\%\;\text{Relative Cytotoxicity}\;=(\frac{\text{ODsample – ODnegative control}}{\text{ODpositive control – ODnegative control}})\times 100$$

### 2.3 Flow Cytometry Analysis

For flow cytometry‐assisted analysis, 5 × 10^5^ cells were plated in 6‐well plates. To assess Annexin V and 7‐AAD assays, the next day the cells were treated with: vehicle or 0.1, 1, 10, 100, or 1000 µM DMI. At 24 h after treatment, the cells were washed and resuspended in 100 μL of binding buffer. For Annexin V and 7‐AAD assays were performed using a commercially available kit (Cat. no. 640922, BioLegend). The reagents were added to the cell suspension, which was then incubated for 15 min at room temperature in the dark, following the manufacturer's instructions. After incubation, 400 μL of binding buffer was added, and the cell preparation was analysed by flow cytometry (MACSQuant Analyzer 16, Miltenyi Biotec). To measure mitochondrial ROS levels, cells were treated with vehicle, 10, or 1000 µM DMI the day after plating. At 24 h after treatment, the cells were stained with 2.5 μM of MitoSOX probe (Cat. no. M36008, Invitrogen) for 30 min at 37°C, and the flow cytometry‐assisted analysis was assessed through the MACSQuant Analyser 16 (Miltenyi Biotec). Data were analysed using FlowJo Software (BD Biosciences).

### 2.4 Cellular Thermal Shift Assay (CETSA)

NSCs were cultured in T75 flasks under standard conditions (37°C, 5% CO₂) for 4 days as spheres. Cells were then collected, centrifuged at 300*g* for 5 min, and washed in PBS. After centrifugation, supernatants were removed, and proteins were extracted with RIPA buffer (Cat. no. ab156034, Abcam) mixed with protease inhibitor (Cat. no. P8340, Sigma‐Aldrich, 1:100). Equal amounts of protein were transferred to new tubes, and vehicle (i.e., DMSO) or 1000 μM DMI in DMSO were added to the samples and incubated for 1 h at 37°C. Subsequently, samples were heated for 3.5 min at either 68°C, 72°C, 76°C, or 80°C using a calibrated dry block heater. After heating, samples were centrifuged at 15,000*g* for 20 min at 4°C to pellet thermally aggregated proteins. Supernatants containing thermally stable soluble proteins were electrophoresed at 160 V for 1 h at room temperature and then transferred to nitrocellulose membranes at 300 mA for 3 min. Membranes were then blocked for 1 h at room temperature with 5% non‐fat dried milk in PBS containing 0.1% Tween‐20, and incubated overnight at 4°C with mouse anti‐Sdha primary antibody (Cat. no. ab14715, Abcam, RRID: AB_301433, 1:1000).

The following day, membranes were washed 3 times for 5 min with 0.1% Tween‐20 in PBS at room temperature and then incubated with anti‐mouse secondary antibody HRP (Cat. no. 31430, Invitrogen, 1:5000) for 1 h at room temperature.

Protein bands were detected by ECL (Cat. no. 34580, Thermo Scientific) and imaged using a Bio‐Rad ChemiDoc MP system. ImageJ software was used to quantify the density of each protein band and expressed as fold change (FC) relative to the optical density of the control band at 68°C (Vicario et al. 2019).

### 2.5 Extracellular Flux (XF) Analysis and Mitochondrial Stress Test

For the XF assay, undifferentiated NSCs were dissociated and seeded into a 24‐well Seahorse XF Cell Culture Microplate coated with laminin (1%, Cat. no. 11243217001, Roche) at a final density of 3 × 10^5^ cells per cm^2^ in complete growth medium. Sodium succinate (500 µM, Cat. no. 483554, Carlo Erba) and/or DMI (10 µM, Cat. no. 592498, Sigma‐Aldrich) were added to cell cultures for 24 h. Control cultures received the corresponding vehicles (PBS and/or DMSO) in equal volumes. After 24 h treatment, culture medium was removed, cells were washed with sterile PBS, and Seahorse XF DMEM assay medium, supplemented and adjusted to pH 7.35–7.45, was added. Plates were incubated for 1 h at 37°C in a non‐CO₂ incubator to allow temperature and pH equilibration. Mitochondrial function was then evaluated using the Seahorse XF Cell Mito Stress Test Kit (Cat No.03016‐100, Agilent) according to the manufacturer's instructions. Briefly, sequential injections of 1.5 µM oligomycin, 1 µM FCCP, and 0.5 µM rotenone/antimycin A were used, and the mitochondrial respiratory parameters oxygen consumption rate (OCR) and extracellular acidification rate (ECAR) were measured using the Seahorse XFe24 Analyzer. Data were normalised to the total protein content per well and are expressed as pmol/min/μg for OCR and mpH/min/μg for ECAR.

### 2.6 Interaction Network and Gene Ontology (GO) Analysis

The interaction network of Cxs and the itaconate signalling pathway was obtained using the STRING database, followed by GO enrichment analysis (https://string-db.org/, accessed August, 2025). The input genes related to Cxs (i.e., Cxs query) were: Gjb2 (encoding Cx26), Gjc1 (encoding Cx45), Gja1 (encoding Cx43), Gjb1 (encoding Cx32), and Gjd2 (encoding Cx36). The input genes related to itaconate signalling (i.e., itaconate signalling query) were Akt1, Nfe2l2, Nfkbie, Sdha, and Tfeb. The minimum required interaction score was 0.150, with a maximum of 25 interactors in the first and second shells of interaction. Edge thickness reflects the confidence of the predicted interaction based on STRING evidence and data support, including text mining, experiments, databases, co‐expression, neighbourhood, gene fusion, and co‐occurrence. Functional enrichment analyses of cellular components, biological processes, and molecular functions were performed using the STRING GO tool. For enrichment analysis, terms were grouped by similarity ≥ 0.8, with the number of genes represented by circle size and sorted by signal.

### 2.7 Immunoblotting

For western blot analysis, cells were plated at a final density of 100,000 cells/cm^2^. After 3 h, DMI at a final concentration of 10 μM, or an equal amount of vehicle, was added to cell cultures. After 24 h, pellets from each sample were collected, centrifuged, and washed with PBS. Supernatants were removed, and proteins were extracted with RIPA buffer (Cat. no. ab156034, Abcam) mixed with protease inhibitor (Cat. no. P8340, Sigma‐Aldrich, 1:100). Equal amounts of protein were electrophoresed at 160 V for 1 h at room temperature and then transferred to nitrocellulose membranes at 300 mA for 3 min. Membranes were then blocked for 1 h at room temperature with 5% non‐fat dried milk (Cat. no. A0830, PanReac AppliChem) in PBS containing 0.1% Tween‐20, and incubated overnight at 4°C with the following primary antibodies diluted in blocking buffer: rabbit anti‐Sox2 (Cat. no. ab97959, Abcam, RRID: AB_2341193, 1:1000), rabbit anti‐Cx26 antibody (Cat. no. ab65969, Abcam, RRID: AB_1140895, 1:500), rabbit anti‐Cx45 antibody (Cat. no. 40‐7000, Thermo Fisher Scientific, RRID: AB_2533479, 1:1000), rabbit anti‐Cx32 antibody (Cat. no. 710600, Invitrogen, RRID: AB_2533972, 1:1000); mouse anti‐Cx36 antibody (Cat. no. 374600, Invitrogen, RRID: AB_2533320, 1:1000); rabbit anti‐Cx43 antibody (Cat. no. C6219, Sigma‐Aldrich, RRID: AB_476857, 1:1000); rabbit anti‐Akt antibody (Cat. no. 9272S Cell Signaling, RRID: AB_329827, 1:1000); rabbit anti‐p‐Akt antibody (Cat. no. 4058S, Cell Signaling, RRID: AB_331168, 1:1000); mouse anti‐β‐actin antibody (Cat. no. sc47778, Santa Cruz Biotechnology, RRID: AB_626632, 1:1000). The following day, membranes were washed in 0.1% Tween‐20 in PBS 3 times for 5 min at room temperature and then incubated with the following secondary antibodies: anti‐rabbit monoclonal antibody HRP (Cat. no. 31460, Invitrogen, RRID: AB_228341, 1:10000) or anti‐mouse monoclonal antibody HRP (Cat. no. 31430, Invitrogen, RRID: AB_228307, 1:5000). Between each primary antibody incubation, a stripping procedure was performed. Membranes were washed in 0.1% Tween‐20 in PBS and incubated with a homemade stripping buffer (6.25% Tris‐HCl pH = 6.8, 2% SDS, 0.7% β‐mercaptoethanol in H_2_O) at room temperature for 1 h. After 3 washes with 0.1% Tween 20 in PBS, membranes were incubated with blocking buffer, followed by a new cycle of primary and secondary antibodies. Protein bands were detected by ECL and imaged using a Bio‐Rad ChemiDoc MP system. ImageJ software was used to quantify the density of each protein band, normalised to β‐actin optical density measured on the same membrane.

### 2.8 Immunofluorescence

For immunofluorescence staining of NSCs as spheres, cells were cultured under standard culture conditions as described above, and when neurospheres reached a diameter of about 100 μm, they were collected and seeded on a Matrigel‐coated 13 mm glass coverslip. After 1 h, neurospheres were fixed in 4% PFA in PBS with 2% sucrose (Cat. no. S0389, Sigma‐Aldrich) for 10 min at room temperature.

For immunofluorescence staining of adherent NSCs, a single cell suspension was plated at a final density of 100,000 cells per glass coverslip, in complete growth medium supplemented with 1% laminin (Cat. no. 11243217001, Roche). The following day, cells were exposed to DMI diluted in growth medium at a final concentration of 10 μM (as specified in the figure legend) and incubated for 24 h. For DMI treatment, a 25 mM stock solution of DMI was prepared in DMSO, and DMI was added to cultures at a maximum concentration of 1.0%. Control groups received an equal volume of DMSO. After 24 h, cells were fixed in 4% PFA in PBS with 2% sucrose for 10 min at room temperature.

For differentiated NSCs, treatments with DMI (10 μM), carbenoxolone (CBX, Cat. no. C4790, Sigma Aldrich, 10 μM) (Vicario et al. 2017), and/or mefloquine (Cat. no. M2319, Sigma Aldrich, 1 μM) (Cruikshank et al. 2004) were added at Day 0 and at Day 3. After 6 days, cells were fixed in 4% PFA in PBS with 2% sucrose for 10 min at room temperature.

Samples were blocked in a solution composed of 10% normal goat serum (NGS, Cat. no. ab7481, Abcam) and 0.3% Triton X‐100 in PBS, for 1 h at room temperature and incubated overnight at 4°C with the following primary antibodies: rabbit anti‐Sox2 (Cat. no. ab97959, Abcam, RRID: AB_2341193, 1:100), mouse anti‐Nestin (Cat. no. MAB11004, Merck, RRID: AB_2536821, 1:100), rabbit anti‐Gfap polyclonal antibody (Cat. no. sab2702475, Sigma‐Aldrich, RRID: n.a., 1:100), mouse anti‐β‐tubulin class III (Tuj1) monoclonal antibody (Cat. no. 801201, Biolegend, RRID: AB_2313773, 1:100), mouse anti‐oligodendrocytes marker (Olm) monoclonal antibody (Cat. no. mab1580, Sigma‐Aldrich, RRID: AB_11213204, 1:100), rabbit Cx32 antibody (Cat. no. 710600, Invitrogen, RRID: AB_2533972, 1:100), mouse Cx36 antibody (Cat. no. 374600, Invitrogen, RRID: AB_2533320, 1:100), and/or rabbit Cx43 antibody (Cat. no. 3512S, Cell Signaling, RRID: AB_2294590, 1:100).

The following day, samples were washed 3 times with 0.3% Triton X‐100 in PBS for 5 min and incubated for 1 h at room temperature with the following secondary antibodies: goat anti‐mouse polyclonal antibody Alexa Fluor 546 (Cat. no. A11003, Invitrogen, RRID: AB_141370, 1:1000) and goat anti‐rabbit polyclonal antibody Alexa Fluor 488 (Cat. no. A11008, Invitrogen, RRID: AB_143165, 1:1000). After 3 washes in 0.3% Triton X‐100 in PBS for 5 min, nuclei were counterstained with 4’,6‐diamidino‐2‐phenylindole (DAPI, Cat. no. D1306, Invitrogen, 1:1000) for 3 min at room temperature and then mounted with Fluoromount Aqueous Mounting Medium (Cat. no. F4680, Sigma‐Aldrich). Digital images were acquired using a Leica TCS SP8 confocal microscope, and images were analysed using ImageJ software.

The quantification of marker‐positive cells was performed by 2 independent operators blinded to the treatment groups, counting the total number of Dapi‐positive cells and the number of Gfap‐, Olm‐, or Tuj1‐positive cells. Data are expressed as mean percentage ± SEM from *n* ≥ 5 independent biological replicates, with a total number of *n* ≥ 139 cells analysed per group. The quantification of mean fluorescence intensity (MFI) of Cx32, Cx43, and Cx36 was performed by two independent operators blinded to the treatment groups by quantifying the fluorescence intensity of *n* = 4 independent biological replicates per group. The fluorescence intensity was divided by the area and expressed as MFI ± SEM.

### 2.9 Dye Transfer Assay and Cell Coupling

Cell coupling experiments were performed using a dye‐transfer assay based on calcein and Dil labelling followed by live‐cell imaging. NSCs were dissociated and resuspended at a final density of 1 × 10^6^ cells per ml in complete growth medium. For calcein labelling, NSCs were incubated with calcein‐AM green (Cat. no. C34852, Invitrogen 0.5 µM) for 30 min at 37°C. Matched NSCs samples were stained with Dil red (Cat. no. SC391095, Santa Cruz, 1.0 µg/ml) for 20 min at 37°C. Samples were then washed with PBS, centrifuged, and resuspended in complete growth medium supplemented with laminin (Cat. no. 11243217001, Roche, 1:100). Calcein‐labelled and Dil‐labelled cells were mixed 1:1 and seeded into a 96‐well plate at a final density of 3 × 10^5^ cells per cm^2^. Cells were treated with 10 µM DMI (Cat. no. 592498, Sigma‐Aldrich), 10 µM DMI + 10 µM CBX (Cat. no. C4790, Sigma Aldrich), or 10 µM DMI + 1 µM mefloquine (Cat. no. M2319, Sigma Aldrich). GJ–mediated dye transfer was monitored by live‐cell imaging using a Cytation 5 imaging system (Agilent) for 180 min. Quantification of calcein and Dil colocalisation was performed at 0, 60, 120, and 180 min by quantifying the fluorescence intensity of *n* = 4 independent biological replicates per group. The colocalisation index was normalised over the average colocalisation at 0 min and data are shown as relative cell coupling mean ± SEM. Linear regression analysis, curve equations, and 95% confidence intervals were calculated using the relative cell coupling.

### 2.10 Statistical Analysis

All statistical tests were performed using GraphPad Prism (v. 5.00 for Mac, GraphPad Software, San Diego, CA, USA).

Data were tested for normality using the Shapiro‐Wilk normality test and subsequently assessed for homogeneity of variance. Data that passed both tests were further analysed by two‐tailed unpaired Student's *t*‐test for comparison between two groups. Comparisons of *n* > 2 groups were performed using a one‐way ANOVA and Holm‐Sidak's multiple comparisons test. Data that did not pass the normality test were assessed using the Mann‐Whitney test for *n* = 2 groups or the Kruskal−Wallis test with multiple comparisons for *n* > 2 groups. For all statistical tests, *p* values < 0.05 were considered statistically significant; *p* values and the number of independent biological replicates are reported in the figures and figure legends.

## 3 Results

### 3.1 Effect of DMI on Viability and Metabolic Turnover of NSCs

**Figure 1:**
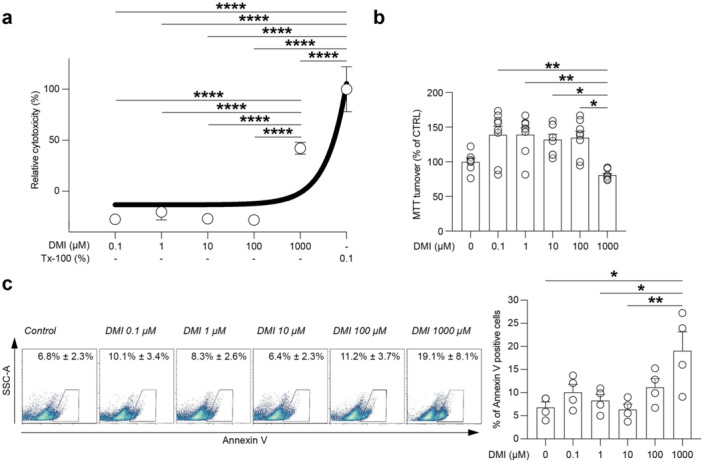
LDH and MTT assay on NSCs treated with increasing concentrations of DMI. (a) LDH viability assay on NSCs cultures treated with 0.1, 1, 10, 100, or 1000 µM of DMI at 24 h. Data are expressed as relative cytotoxicity (%) normalised to vehicle‐treated control (0%) and 0.1% of Tx‐100‐treated cultures (100%) mean ± SEM of *n* ≥ 6 independent replicates. (b) MTT turnover of NSCs cultures treated with 0.1, 1, 10, 100, or 1000 µM of DMI at 24 h. Data are expressed as a % of control (vehicle‐treated cultures, 100%) and shown as dot plots and mean ± SEM of *n* ≥ 7 independent replicates. (c) Cytofluorimetric analysis of annexin V‐positive cells of NSCs cultures treated with 0, 0.1, 1, 10, 100, or 1000 µM of DMI at 24 h. Data are shown as a % of gated cells and shown as dot plots and mean ± SEM of *n* = 4 independent replicates. *p value < 0.05, **p value < 0.01, ****p value < 0.0001.

We first characterised SVZ‐derived NSCs in vitro by analysing the expression of typical markers, growing NSCs as monolayers or neurospheres (Supporting Information S1: Figure 1a). Cells were stained by immunofluorescence to assess the expression of Sox2 and Nestin, and were found positive for NSCs markers. Cultures were also characterised over consecutive passages to quantify the total number of cells, starting from a plating number of 2.5 × 10^5^ cells (passage 0) to a total number of about 7.06 × 10^8^ ± 1.21 × 10^8^ cells after five consecutive passages (Supporting Information S1: Figure 1b). The R.C.G. was also calculated (3.48 ± 0.34 passage 1, 4.75 ± 0.45 passage 2, 5.09 ± 0.49 passage 3, 5.64 ± 0.65 passage 4, and 6.16 ± 0.33 passage 5, Supporting Information S1: Figure 1c), showing a stable and expandable potential of the derived cultures.

**Figure 2:**
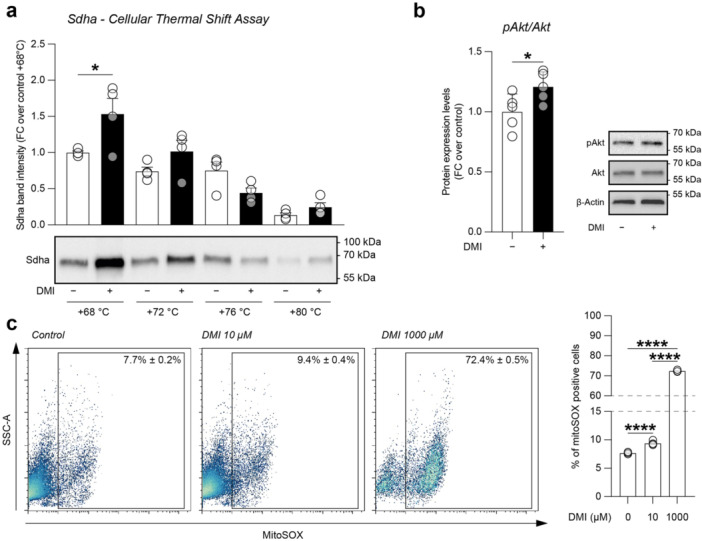
Effect of DMI on Shda, pAkt/Akt, and redox balance in NSCs. (a) Representative blot from a CETSA showing Sdha‐1000 μM DMI interactions at 4 temperatures from +68°C to +80°C. (b) Quantification and representative blots of pAkt/Akt protein expression levels in control (vehicle‐treated) and 10 μM DMI‐treated NSCs after 24 h. Data are shown as dot plots and mean ± SEM of n ≥ 4 independent replicates. (c) Cytofluorimetric analysis of mitoSOX‐positive cells of NSCs cultures treated with 0, 10, or 1000 µM of DMI at 24 h. Data are shown as a % of gated cells and shown as dot plots and mean ± SEM of *n* = 4 independent replicates. *p value < 0.05 and ****p value < 0.0001.

We then evaluated the effect of increasing concentration of DMI in vitro, assessing both its potential cytotoxicity and metabolic impact on NSCs, starting from intracellular concentration typically reported in basal conditions up to 1000 µM. The LDH viability assay showed that, at concentrations ranging from 0.1 to 100 µM, DMI did not significantly increase the relative cytotoxicity as compared to untreated control cultures (Figure 1a). Importantly, DMI at 1000 µM concentration significantly increased relative cytotoxicity compared to all other tested concentrations (42.19% ± 2.07% for DMI 1000 µM, *p*‐value < 0.0001 vs. 0.1, 1, 10, and 100 µM; Figure 1a). Potential interference between the LDH assay and DMI was assessed using complete growth medium without cells and complete growth medium containing 1000 µM of DMI, obtaining comparable results (Supporting Information S1: Figure 2a). We then analysed the effect of DMI on the metabolic turnover/mitochondrial integrity upon treatment using an MTT assay (Figure 1b). A slight, non‐significant increase in MTT turnover was observed in cultures treated with 0.1 µM (139.0% ± 12.41%, *p* value = 0.37), 1 µM (139.2% ± 9.78%, *p* value = 0.37), 10 µM (132.2% ± 7.73%, *p* value > 0.99) and 100 µM (134.8% ± 9.18%, *p*‐value = 0.86) of DMI concentrations as compared to the control (100.0% ± 5.28%, Figure 1b). Treatment with 1000 µM of DMI, even if it lowered the MTT turnover by about 20%, was not significantly reduced as compared to control at 24 h post‐DMI treatment (80.77% ± 2.42% 1000 µM DMI vs. 100.0% ± 5.28% 0 µM DMI, *p* value > 0.99). Interestingly, 1000 µM DMI induced a significant reduction of the metabolic activity in NSCs as compared to 0.1 µM (*p* value = 0.00287), 1 µM (*p* value = 0.00287), 10 µM (*p* value = 0.01591), and 100 µM (*p* value = 0.01147; Figure 1b).

To confirm the effect of increasing concentrations of DMI, we performed a cytofluorimetric analysis of early apoptotic cells stained with annexin V. At 24 h, we found that 1000 µM DMI induced an increase in the proportion of annexin V‐positive cells as compared to control (19.1% ± 4.1% for DMI 1000 µM vs. 6.8% ± 1.2% for control, *p* value = 0.0103; Figure 1c). No significant changes were observed at lower concentrations of DMI in the proportion of annexin V‐positive cells at 24 h post‐treatment (Figure 1c). We further quantified late apoptotic/necrotic cells and found that after 24 h of DMI treatment, no significant changes in the proportion of 7‐AAD‐positive cells were detected at the tested time point (Supporting Information S1: Figure 2b).

### 3.2 Intracellular Signalling and Metabolic Reshaping Mediated by DMI on NSCs

**Figure 3:**
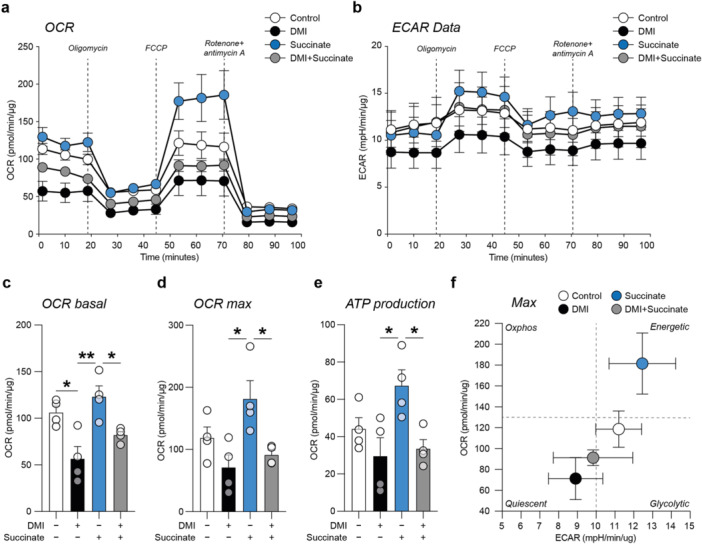
Oxygen consumption rate and extracellular acidification rate of DMI‐ and succinate‐treated NSCs. (a, b) Extracellular flux (XF) assay of the OCR (a) and ECAR (b) in control NSCs and NSCs treated with 10 μM DMI, 500 μM succinate or 10 μM DMI + 500 μM succinate during sequential injections of 1.5 µM oligomycin, 1 µM FCCP, and 0.5 µM rotenone/antimycin A. (c−e) Normalised basal OCR (c), maximal OCR (d), and ATP production (e) in control NSCs and NSCs treated with 10 μM DMI, 500 μM succinate or 10 μM DMI + 500 μM succinate. (f) Biplot of maximal OCR and ECAR for control NSCs and NSCs treated with 10 μM DMI, 500 μM succinate or 10 μM DMI + 500 μM succinate. Data are expressed as mean ± SEM of *n* = 4 replicates. *p value < 0.05 and **p value < 0.01.

To evaluate the potential capability of DMI to activate intracellular mediators, we performed a CETSA on Sdha (Figure 2a, Supporting Information S1: Figure 3a). Our data revealed a significant increase in Sdha stability at 68°C compared to control (1.53 ± 0.21 vs. 1.00 ± 0.02, *p*‐value = 0.04816, Figure 2a), indicating a ligand‐binding‐induced stabilisation mediated by DMI. Given the importance of the Akt signalling pathway in both stem cell fate and itaconate‐mediated signalling, we performed a western blot analysis on the levels of phosphorylated Akt (Ser473, Figure 2b, Supporting Information S1: Figure 3b). Our analysis revealed that DMI treatment increased pAkt levels, suggesting DMI‐mediated activation of the Akt signalling pathway (1.27 ± 0.16 vs. 1.00 ± 0.07, *p*‐value = 0.0455; Figure 2b).

Given the metabolic role of Sdha and the potential role of DMI in modulating cellular metabolism and redox balance, we performed mitoSOX staining on NSCs exposed to 10 or 1000 µM DMI for 24 h (Figure 2c). Our data revealed that 1000 µM of DMI markedly increased the proportion of mitoSOX‐positive cells (72.4% ± 0.5% 1000 µM DMI vs. 7.7% ± 0.2% control, *p* value < 0.0001; Figure 2c), whereas 10 µM of DMI induced a minor increase in the proportion of mitoSOX‐positive cells (9.4% ± 0.4% 10 µM DMI, *p* value < 0.0001 10 µM DMI vs. control and *p* value < 0.0001 10 µM DMI vs. 1000 µM DMI; Figure 2c), consistent with their potential role as signalling molecules. Thus, 10 µM DMI was used for subsequent analysis.

To link the molecular effects of DMI with functional metabolic reshaping, we performed an XF assay of the OCR (Figure 3a) and ECAR (Figure 3b) during a mitochondrial stress test. We compared DMI‐treated NSCs with succinate‐ and DMI + succinate‐treated cultures to evaluate the ability of DMI to functionally inhibit mitochondrial respiration.

Our results showed that DMI strongly reduces basal OCR compared to control (56.66 ± 13.07 pmol/min/µg DMI vs. 106.27 ± 6.80 pmol/min/µg control, *p* value = 0.0156 DMI vs. control; Figure 3c) and to succinate‐treated NSCs (123.24 ± 11.50 pmol/min/µg succinate, *p* value = 0.0021 DMI vs. succinate; Figure 3c). We also observed a significantly lower basal OCR in DMI + succinate‐treated cells compared to succinate alone (82.09 ± 3.77 pmol/min/µg DMI + succinate, *p* value = 0.0396 DMI + succinate vs. succinate; Figure 3c). Moreover, our data on maximal OCR showed that DMI significantly reduces maximal OCR compared to succinate‐treated cells (71.28 ± 20.13 pmol/min/µg DMI and 181.47 ± 29.33 pmol/min/µg succinate, *p* value = 0.0135 DMI vs. succinate; Figure 3d), with a significant reduction of maximal OCR also observed in DMI+succinate‐treated cell cultures (91.36 ± 7.56 pmol/min/µg DMI+succinate, *p* value = 0.0406 DMI + succinate vs. succinate; Figure 3d).

Similar results were observed in the ATP production, showing significant lower levels in DMI‐treated cells alone or in combination with succinate (29.61 ± 9.84 pmol/min/µg DMI and 33.60 ± 4.79 pmol/min/µg DMI + succinate; Figure 3e), compared with succinate‐treated cultures (67.36 ± 8.45 pmol/min/µg succinate, *p*‐value = 0.0241 DMI vs. succinate, and *p* value = 0.0398 DMI + succinate vs. succinate; Figure 3e).

Biplot analysis of OCR and ECAR showed that succinate increases the energetic profile of NSCs and that DMI induced a reshaping of the metabolic profile toward a quiescent phenotype compared to control NSCs cultures (Figure 3f). Collectively, these data support the hypothesis that DMI suppresses mitochondrial oxidative metabolism and electron transport chain, consistent with Sdha inhibition, preventing succinate‐induced stimulation of respiration and shifting NSCs towards a low‐energy, quiescent metabolic state.

### 3.3 Modulation of Cxs Expression Profile and Function Induced by DMI

**Figure 4:**
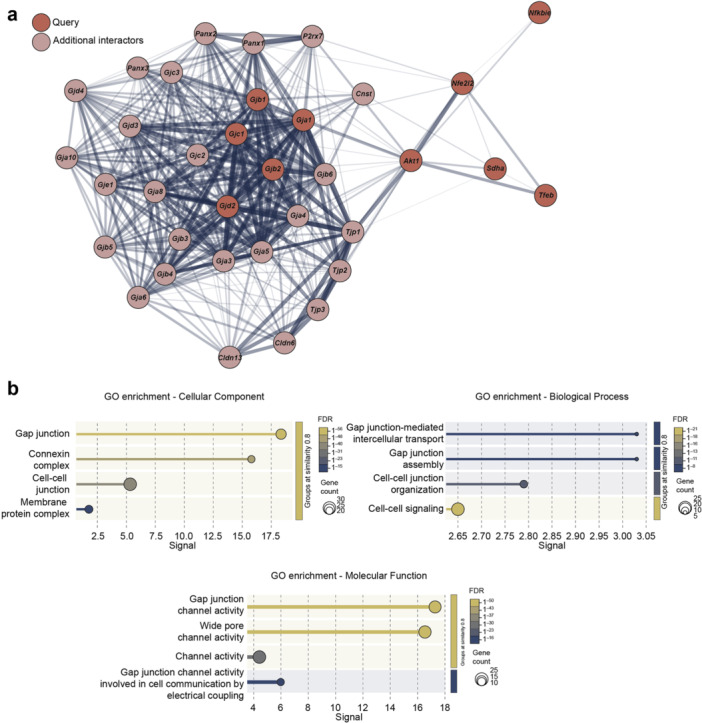
Interaction between connexins and the itaconate signalling pathway. (a) Interaction network of 10 selected genes (i.e., query) and the top additional interactor genes; interactions are shown based on databases or experimentally determined interactions. (b) Gene Ontology (GO) enrichment analysis of genes for cellular component, biological processes, and molecular function related to Cxs and itaconate signalling. Data are reported as a function of the signal. Dots' dimension and colour are related to the total number of gene counts involved in the pathway and the false discovery rate (FDR), respectively.

To evaluate the potential interplay between Cxs and itaconate‐mediated effects on NSCs, we performed an interaction network analysis of Cxs expressed in undifferentiated and differentiated NSCs along with DMI signalling, based on available databases and experimentally determined interactions (Figure 4a). Our analysis revealed a strong interaction network between Cxs genes, namely Gjb2, which encodes Cx26, Gjc1, which encodes Cx45, Gja1, which encodes Cx43, Gjb1, which encodes Cx32, and Gjd2, which encodes Cx36, and itaconate‐mediated signalling, and a strong interaction between Gja1 and Akt1 signalling pathways (Figure 4a).

We also performed a GO enrichment analysis for cellular component, biological process, and molecular function (Figure 4b) involved in the interaction network. We found that among the most significant cellular components “Gap junction” (–Log_10_FDR = 55.49 and signal = 18.35) and “Connexin complex” (–Log_10_FDR = 44.75 and signal = 15.79) were strongly represented (Figure 4b), and “Gap junction‐mediated intercellular transport” (–Log_10_FDR = 7.92 and signal = 3.03), “Gap junction assembly” (–Log_10_FDR = 7.92 and signal = 3.03) and “Cell‐cell signaling” (–Log_10_FDR = 21.62 and signal = 2.65) were among the most enriched biological processes (Figure 4b). GO enrichment of molecular function highlighted “Gap junction channel activity” and “Wide pore channel activity” as the most represented pathways (–Log_10_FDR = 49.53 and signal = 17.28 and –Log_10_FDR = 49.53 and signal = 16.54; Figure 4b).

**Figure 5:**
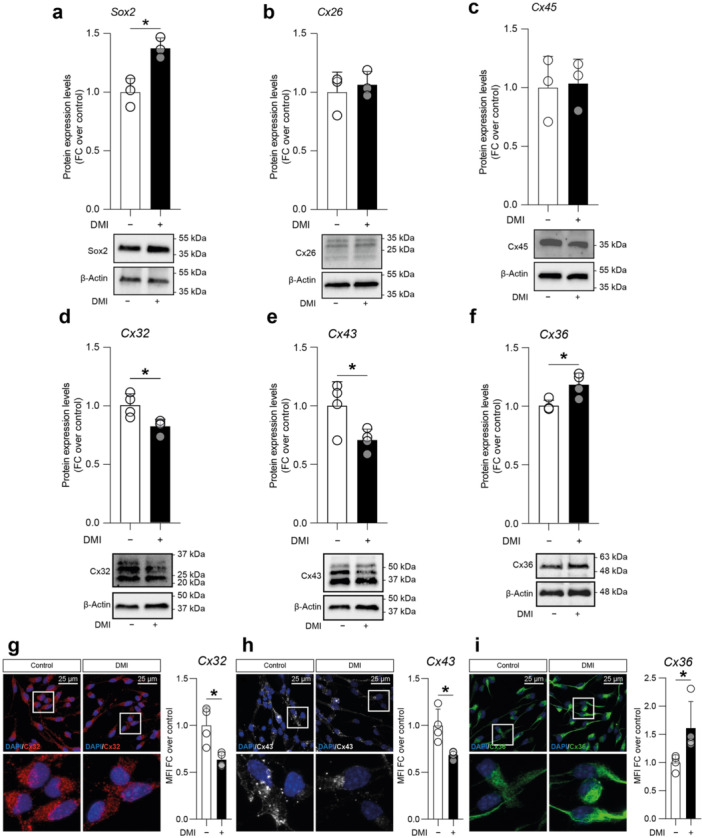
Expression levels of Sox2, Cx26, Cx45, Cx32, Cx43, and Cx36 in NSCs treated with DMI. (a−f) Quantification and representative blots of Sox2 (a), Cx26 (b), Cx45 (c), Cx32 (d), Cx43 (e), and Cx36 (f) protein expression levels in control (vehicle treated) and 10 μM DMI‐treated NSCs after 24 h. (g−i) Representative confocal microscopy images and quantification of the mean fluorescence intensity (MFI) of undifferentiated NSCs in the control condition or treated with 10 μM DMI and stained for Cx32 (red, g), Cx43 (white, h), and Cx36 (green, i). Nuclei were counterstained with Dapi (blue, g−i). Data are shown as dot plots and mean ± SEM of *n* ≥ 3 independent replicates. *p value < 0.05.

To characterise the molecular dynamics induced by DMI treatment, we analysed the protein expression levels of Sox2, as well as Cx26 and Cx45, which are typically expressed by undifferentiated NSCs (Figure 5a−c, Supporting Information S1: Figure 4). We found that DMI treatment significantly increased Sox2 expression compared to control cultures (1.38 ± 0.09 vs. 1.00 ± 0.12, *p* value = 0.0114; Figure 5a), indicating enhanced stemness and retained NSCs multipotency. Interestingly, Cx26 and Cx45 expression levels were not significantly altered by DMI treatment at 24 h (1.00 ± 0.17 vs. 1.07 ± 0.11 for Cx26, *p* value = 0.6104; Figure 5b; 1.00 ± 0.27 vs. 1.04 ± 0.21 for Cx45, *p* value = 0.8619; Figure 5c), aligning with intercellular coupling typically observed in stem cell niches.

We also analysed the expression levels of Cxs typically associated with oligodendrocytes (Cx32), astrocytes (Cx43), and neurons (Cx36) of control and DMI‐treated undifferentiated NSCs (Figure 5 and Supporting Information S1: Figure 5). Our analysis revealed that DMI treatment significantly reduced Cx32 (0.82 ± 0.03 vs. 1.00 ± 0.05, *p* value = 0.0205; Figure 5d) and Cx43 (0.71 ± 0.05 vs. 1.00 ± 0.10, *p* value = 0.0417; Figure 5e) expression levels as compared to controls (Figures 5d,e), whereas a significant increase in neuronal‐associated Cx36 was observed in DMI‐exposed cultures (1.18 ± 0.05 vs. 1.00 ± 0.02, *p* value = 0.0168; Figure 5f). We confirm this modulatory effect of DMI on Cxs expression by confocal‐assisted imaging on undifferentiated NSCs, finding a significant reduction of Cx32 (0.64 ± 0.04 vs. 1.00 ± 0.10, *p* value = 0.01355; Figure 5g) and Cx43 MFI (0.69 ± 0.02 vs. 1.00 ± 0.09, *p* value = 0.01333; Figure 5h), coupled with a significant increase of Cx36 MFI (1.62 ± 0.23 vs. 1.00 ± 0.07, *p* value = 0.02857; Figure 5i).

**Figure 6:**
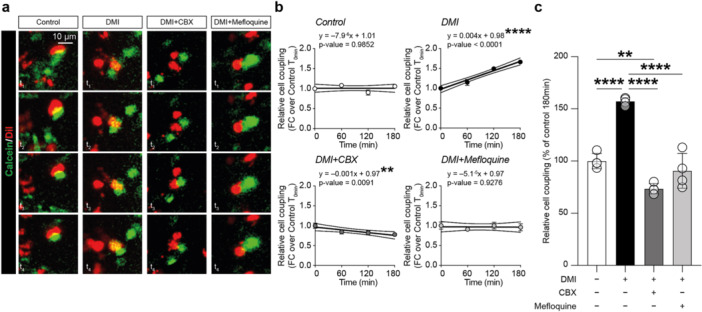
Modulatory effect of DMI on GJs intercellular communication in NSCs. (a) Live imaging of control NSCs and NSCs treated with 10 μM DMI, 10 μM DMI + 10 μM CBX, and 10 μM DMI + 1 μM mefloquine stained with calcein (green) or Dil (red) and cocultured. T_1_−T_4_ refer to consecutive images. (b) Linear regression analysis and curves’ equations of relative cell coupling measured at 0, 60, 120 and 180 min post‐culture normalised over 0 min in control NSCs and NSCs treated with 10 μM DMI, 10 μM DMI + 10 μM CBX, and 10 μM DMI + 1 μM mefloquine. The central line represents the interpolation, and 95% confidence interval is shown above and below. c) Relative cell coupling in control NSCs and NSCs treated with 10 μM DMI, 10 μM DMI + 10 μM CBX, and 10 μM DMI + 1 μM mefloquine. Data are shown as mean ± SEM of *n* = 4 independent replicates. **p value < 0.01 and ****p value < 0.0001.

In an effort to link changes in Cxs protein levels with a potential increase of GJs intercellular communication, we performed a dye‐coupling assay between NSCs, following DMI treatment, using the non‐selective GJs inhibitor CBX or the selective Cx36‐based GJs inhibitor mefloquine (Figure 6a). Our data revealed that DMI‐treated NSCs showed a significant positive linear correlation between relative cell coupling and time (*p* value = 0.0001; Figure 6b). Interestingly, while control and DMI+mefloquine showed no significant changes of cell coupling over time, a negative correlation was observed in DMI + CBX (Figure 6b), likely due to the non‐selective inhibition of GJIC.

Quantification of the relative cell coupling at 180 min post‐treatment showed an increase in DMI‐treated NSCs compared to control cultures (157.24% ± 1.77% DMI and 100.00% ± 3.66% control, *p* value < 0.0001 DMI vs. control; Figure 6c). Interestingly, CBX in combination with DMI abolished cell coupling with a slight decrease of the relative cell coupling (73.51% ± 2.49% DMI + CBX, *p* value < 0.0001 DMI + CBX vs. control; Figure 6c), indicating an inhibition of intercellular communication mediated by CBX, whereas the selective inhibitor of Cx36‐based GJ mefloquine, fully reverted DMI‐induced effects at the level of controls (90.62% ± 8.39% DMI+mefloquine, *p* value < 0.0001 DMI‐mefloquine vs. DMI, and *p* value = 0.1942 DMI‐mefloquine vs. control; Figure 6c). Taken together, these data corroborate the hypothesis of a prominent role of Cx36‐based intercellular coupling in DMI‐treated NSCs cultures.

### 3.4 Differentiation Profile of NSCs Treated With DMI

**Figure 7:**
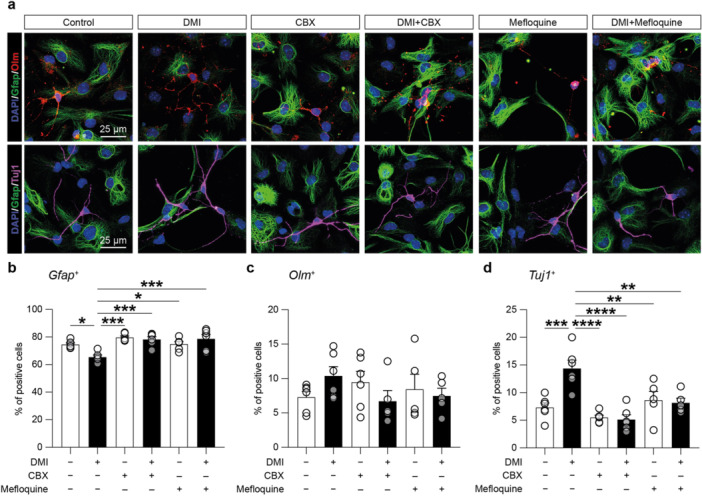
Effect of DMI and GJs modulators on NSCs differentiation profile. (a) Representative confocal microscopy images of NSCs differentiated in control or treated with 10 μM DMI, 10 μM CBX, 10 μM CBX + 10 μM DMI, 1 μM mefloquine, or 1 μM mefloquine+10 μM DMI and stained for Gfap (green) and oligodendrocyte marker (Olm, red; upper panels) and Gfap (green) and Tuj1 (magenta; bottom panels). Nuclei were counterstained with Dapi (blue). (b–d) Quantification of the percentage of Gfap‐ (b), Olm‐ (c) and Tuj1‐(d)‐positive cells over Dapi. Data are shown as dot plots and mean ± SEM of *n* ≥ 5 independent replicates. *p value < 0.05, **p value < 0.01, ***p value < 0.001, and ****p value < 0.0001.

To link these biological processes and potential effects on stem cell fate regulation, we analysed the differentiation potential of NSCs upon DMI treatment using known modulators of GJs‐mediated intercellular communication, namely CBX, a non‐selective inhibitor of GJs and HCs, and mefloquine, a relatively selective inhibitor of Cx36‐based channels (Figure 7a−d). Our data revealed that DMI treatment significantly reduced the proportion of Gfap‐positive cells compared to control cultures (65.56% ± 3.48% DMI vs. 74.65% ± 2.69% control, *p* value = 0.01600; Figure 7b). This effect was coupled with no significant changes in the proportion of oligodendrocyte marker (Olm)‐positive cells (7.31% ± 2.00% DMI vs. 10.39% ± 3.27% control, *p* value = 0.85188; Figure 7c). By contrast, the proportion of Tuj1‐positive cells was significantly increased in DMI‐treated cultures compared to controls, suggesting that DMI modulates NSCs differentiation toward the neuronal lineage (7.32% ± 2.01% DMI vs. 14.41% ± 3.58% control, *p* value = 0.04659; Figure 7d).

The non‐selective GJs/HCs blocker CBX did not affect NSCs differentiation compared to controls but was able to revert DMI‐induced effects on NSCs differentiation fate (Figure 7b−d). We observed comparable percentages of Gfap‐positive cells (79.69% ± 2.71% CBX, 78.37% ± 4.58% CBX + DMI; Figure 7b), Olm‐positive cells (9.48% ± 3.80% CBX, 6.72% ± 3.42% CBX + DMI; Figure 7c), and Tuj1‐positive cells (5.53% ± 0.93% CBX, 5.15% ± 2.07% CBX + DMI; Figure 7d) relative to control cultures. This profile differed significantly from that of DMI‐treated cells, with an increased percentage of Gfap‐positive cells (*p* value = 0.00010 DMI vs. CBX and *p* value = 0.00036 DMI vs. CBX + DMI) and a reduced percentage of Tuj1‐positive cells (*p* value = 0.00001 DMI vs. CBX and *p* value = 0.00001 DMI vs. CBX + DMI), indicating the involvement of Cxs‐based channels in DMI‐induced modulation of NSCs differentiation.

Given the increased levels of Cx36 in DMI‐treated cells, we further tested the role of Cx36‐based channels by using the inhibitor mefloquine. Our results showed that mefloquine alone did not significantly modulate NSCs differentiation compared to controls, but it was able to revert the DMI‐induced effects on Gfap‐ and Tuj1‐positive cells.

Our data revealed similar percentages of Gfap‐positive cells (74.96% ± 4.22% mefloquine, 78.75% ± 7.47% mefloquine+DMI; Figure 7b), Olm‐positive cells (8.44% ± 4.87% mefloquine, 7.50% ± 2.45% mefloquine+DMI, Figure 7c), and Tuj1‐positive cells (11.86% ± 5.76% mefloquine, 9.61% ± 5.99% mefloquine+DMI; Figure 7d) compared to controls when mefloquine was added alone or in combination with DMI. Notably, the proportion of Gfap‐positive cells in mefloquine‐ and mefloquine+DMI‐treated cultures was significantly higher than in DMI‐treated cells (*p* value = 0.01271 DMI vs. mefloquine and *p* value = 0.00026 DMI vs. mefloquine+DMI; Figure 7b). Moreover, mefloquine significantly reduced the percentage of Tuj1‐positive cells compared to DMI‐treated cells (*p* value = 0.0073 DMI vs. mefloquine and *p* value = 0.0034 DMI vs. mefloquine+DMI; Figure 7d), indicating that Cx36‐based channels are involved in the DMI‐induced increase of neurons differentiating from NSCs.

## 4 Discussion

NSCs have been extensively studied for their potential to differentiate into neurons, astrocytes, and oligodendrocytes and for their ability to influence and reshape the CNS microenvironment. Intercellular communication among CNS‐resident cell populations represents one of the most important mechanisms for maintaining homeostasis and supporting compensatory and regenerative processes (Verkhratsky et al. 2025). In this context, metabolic mediators such as itaconate and itaconate‐mediated signalling have attracted increasing interest for their roles as immune modulators and their involvement in the regulation of cell proliferation, survival and differentiation (Li et al. 2020; Paulenda et al. 2025).

Here, we characterised SVZ‐derived NSCs and their response to the cell‐permeable itaconate derivative DMI. On the one hand, the dose‐response analysis highlighted a clear cytotoxicity pattern for high doses of DMI, measured in terms of early and late apoptotic cells and extracellular LDH release. On the other hand, low‐to‐moderate doses of DMI induced potential hormetic and biphasic effects, showing overall viability similar to the untreated control, associated with a sustained MTT turnover and a slight increase in mitoSOX levels. It is critical to note that proliferating NSCs typically maintain slightly elevated ROS levels, and previous studies have shown that inhibition of mitochondrial ROS impairs their differentiation profile (Bustamante‐Barrientos et al. 2025; Torrisi et al. 2025). This is consistent with the role of ROS as signalling molecules in modulating cell fate and aligns with the increased Sox2 levels observed in DMI‐treated NSCs. This evidence is consistent with maintenance of a progenitor‐associated transcriptional program, potentially indicating the acquisition of a primed state upon DMI treatment (Tsaytler et al. 2024).

Such observations prompted us to explore whether DMI modulates mitochondrial activity by analysing the effect of DMI on Sdha. We found a strong ligand‐induced thermal stabilisation of Sdha, supporting the possibility that DMI affects Sdha‐associated metabolic processes and of target‐cell metabolism. To confirm this metabolic effect, we performed an XF assay of OCR and ECAR, showing that DMI can induce metabolic rewiring, interfering with succinate‐supported oxidative metabolism, in line with an impairment of Sdha‐dependent function. This is in accordance with previous findings suggesting that Sdha inhibition by itaconate is a key factor in modulating reactive macrophages (Lampropoulou et al. 2016), although this effect alone does not fully explain the pronounced immunoregulatory effects mediated by cell‐permeable itaconate derivatives and their influence on cell proliferation and differentiation (Bambouskova et al. 2018).

Mechanistically, Sdha inhibition mediated by DMI results in increased mitochondrial ROS, which in turn acts as a signalling molecule activating the Akt pathway. Indeed, we found that NSCs exposed to DMI exhibited increased pAkt signalling. This observation is consistent with recent evidence showing that cell‐permeable itaconate can activate the Nrf2/Sirt3 pathway through Akt phosphorylation, providing mechanistic insights into its antioxidant effects (Hu et al. 2024). Moreover, Sohail et al. tested the effects of itaconate and its cell‐permeable derivatives DMI and 4‐octyl itaconate on dTHP1 cells infected with influenza A virus, finding that itaconate cell‐permeable derivatives may drive a redox signalling cascade via Akt activation, consistent with our observations in NSCs (Sohail et al. 2022). In this context, Akt signalling, together with AMP‐activated protein kinase (Ampk), may act as a central metabolic checkpoint that coordinates redox and energy homeostasis (Han et al. 2018). In addition, previous studies have shown that Ampk activation, together with Ampk‐mediated inhibition of glycogen synthase kinase 3β (Gsk3β), promotes Nrf2 nuclear translocation and the transcription of antioxidant and metabolic enzymes (Joo et al. 2016).

Given that NSCs interact with CNS‐resident cell populations, including microglia, through Cxs‐mediated exchange of small molecules, including ROS, and that Akt and ROS signalling are also regulators of intercellular communication, we investigated whether DMI‐mediated effects could affect Cxs expression levels in NSCs (Le et al. 2014; Rouach et al. 2004; Talaverón et al. 2015). Using a bioinformatic approach, we sought to identify common pathways linking known players in itaconate‐induced signalling, with Cxs genes expressed in undifferentiated and differentiated NSCs. Our analysis revealed a strong association between Cxs and Akt signalling, and enrichment analysis highlighted GJ activity, GJ‐mediated intercellular communication, and cell‐cell signalling as the most prominent enriched pathways. Akt has been reported to regulate Cxs at multiple levels. Indeed, Akt can directly affect Cxs at the mRNA or protein levels by interfering with mRNA stability, Cxs phosphorylation and turnover, GJs assembly, and their membrane trafficking (Bhattacharjee et al. 2009); as well as indirectly through downstream transcription regulators (Allagnat et al. 2013; Hu et al. 2024). In particular, the Akt‐Nrf2 axis represents a key regulator of cellular redox balance (Dai et al. 2020), with significant evidence supporting mechanisms involving Cxs homeostasis (Chen et al. 2017; Fetoni et al. 2018; Liu et al. 2017). Although our study did not directly investigate transcriptional regulation, the increase in pAkt signalling following DMI treatment suggests its involvement in the modulation of Cxs expression and function observed in NSCs.

Consistent with this, analysis of Cxs in undifferentiated NSCs exposed to DMI revealed distinct changes in Cxs expression profiles, with upregulation of Cx36, typically associated with neuronal identity, and downregulation of Cx32 and Cx43 levels, linked to oligodendrocytic and astrocytic lineages, respectively. Functionally, these changes were associated with enhanced intercellular communication, as evidenced by increased cell coupling, in line with the increased levels of Cx36‐based channels. It is worth noting that the expression levels of Cx26 and Cx45, both expressed by SVZ‐derived undifferentiated NSCs, were not significantly affected. This may reflect the roles of these Cxs in NSCs homeostasis and the function of Cx45 as a modulator of transit‐amplifying cells and neuroblasts in the postnatal SVZ (Khodosevich et al. 2012).

Moreover, our analysis of differentiated NSCs showed that DMI treatment reduced the proportion of astrocytes while increasing the output of Tuj1‐positive neurons, consistent with the increased Cx36 levels in undifferentiated DMI‐treated NSCs. We further tested DMI in combination with known modulators of Cxs‐based channels. Data using the non‐selective GJ inhibitor CBX showed no significant alterations in NSCs differentiation profile. Importantly, CBX abolished the DMI‐induced effects on NSCs differentiation, supporting a functional link between DMI signalling and GJ‐forming proteins.

It is worth noticing that CBX is a broad‐spectrum inhibitor of intercellular communication mediated by Cxs‐ and pannexins‐based channels (Dahl et al. 2013; Juszczak and Swiergiel 2009). Therefore, the reversal of DMI‐induced effects observed following CBX cannot be attributed exclusively to Cxs inhibition. Pannexin‐based channels, particularly Panx1, are also expressed in neural cells and hold important roles in intercellular signalling and ATP release within the CNS (Bruzzone et al. 2003; Yeung et al. 2020). Similarly, mefloquine, a relatively selective inhibitor of Cx36‐based channels with minimal nonspecific actions (Cruikshank et al. 2004), produced comparable effects, indicating that functional Cx36 channels contribute to the DMI‐induced modulation of NSCs differentiation. Future studies using complementary approaches and genetic evidence, including Cx36 knockdown or knockout strategies, would help to further clarify the contribution of this Cx to the observed effects of DMI on NSCs differentiation profile. In addition, Cx26, Cx30 and Cx43 are known to play key roles in regulating stem cell niches’ homeostasis by coordinating metabolic coupling, ion balance, and intercellular signalling (Kunze et al. 2009). Previous studies showed that Cx30 and Cx43 knockout diminished cell coupling, and such a phenomenon was associated with decreased proliferation and neurogenesis in adult stem cell niches (Kunze et al. 2009). Accumulating evidence points to a pivotal role of Cx43 in this phenomenon, with mild or minimal effects associated with Cx26 or Cx30 in modulating proliferation and neurogenesis in adults (Kunze et al. 2009; Liebmann et al. 2013; Zhang et al. 2018). Although our work focused on Cx36 as a critical neuronal Cx and on its function within the described DMI‐induced signalling pathway, these findings should be considered within the broader context of intercellular communication in the neurogenic niches, where multiple communication mechanisms likely cooperate to regulate cell fate and metabolism (Sánchez et al. 2020; Sanfilippo et al. 2019; Spitale et al. 2020; Vicario et al. 2022).

It is important to consider that DMI represents a cell‐permeable derivative of itaconate and may not recapitulate the signalling induced by endogenous non‐esterified itaconate (Swain et al. 2020). Differences in membrane permeability, intercellular accumulation, and biological kinetics may lead to distinct effects in terms of magnitude and time required to reach comparable effects, making the use of unmodified itaconate an interesting line of research (Swain et al. 2020). Taking into account the differences between itaconate and its derivatives, the described findings may have potential implications for regenerative strategies in the adult neurogenic niches. During inflammatory conditions, such as ischaemic injury, microglia and infiltrating macrophages can release itaconate and other metabolites able to influence surrounding cells and stem cell niches (Hooftman and O'Neill 2019). Therefore, modulation of metabolic signalling in neuroinflammatory and neurodegenerative disorders represents a critical mechanism to foster endogenous compensatory processes and re‐establish microenvironment homeostasis by regulating Cxs‐mediated intercellular communication (Wang et al. 2026).

Collectively, our findings support the role of DMI as a modulator capable of influencing NSCs fate through mechanisms involving Sdha interaction and Akt signalling, coupled with changes in Cxs expression and a preferential differentiation towards a neuronal phenotype. While cell‐permeable itaconate derivatives represent a promising approach to reshaping the metabolism of inflammatory cells and counteracting disease progression, their ability to modulate Cxs expression patterns and impact on cell fate represents a compelling area for future research.

## Author Contributions

Conceptualization: Simona Denaro, Nunzio Vicario, Rosalba Parenti. Methodology: Simona Denaro, Simona D'Aprile, Anna Gervasi, Filippo Torrisi, Carmela Parenti, Agata Zappalà, Nunzio Vicario, Rosalba Parenti. Investigation: Simona Denaro, Simona Rosa Spina, Simona D'Aprile, Anna Gervasi. Data curation: Simona Denaro, Simona Rosa Spina, Nunzio Vicario. Formal analysis: Simona Denaro, Agata Zappalà, Nunzio Vicario, Rosalba Parenti. Writing – original draft: Simona Denaro, Nunzio Vicario. Writing – review and editing: All authors. Project administration: Simona Denaro, Agata Zappalà, Nunzio Vicario, Rosalba Parenti. All authors read and approved the final manuscript.

## Ethics Statement

The authors have nothing to report.

## Consent

The authors have nothing to report.

## Conflicts of Interest

The authors declare no conflicts of interest.

## Supporting information


**Figure S1:** Characterisation of expression markers and proliferative dynamics in mouse SVZ‐derived NSCs. a) Representative confocal microscopy images of NSCs grown as monolayer (top panels) and neurospheres (bottom panels) expressing Sox2 (magenta) and Nestin (white). Nuclei were counterstained with Dapi (blue). b‐c) Linear growth curve and rate of cell growth (R.C.G.) of NSCs over 5 consecutive passages. Data are expressed as mean ± SEM of n = 8 independent replicates.
**Figure S2:** a) Analysis of the absorbance at 450 nm of the medium+vehicle or the medium+1000 μM of DMI without cells. b) Cytofluorimetric analysis of 7‐AAD‐positive cells of NSCs cultures treated with 0, 0.1, 1, 10, 100 or 1000 μM of DMI at 24 hours. Data are shown as a % of gated cells and shown as dot plots and mean ± SEM of n = 4 independent replicates.
**Figure S3:** Whole uncropped images of the original western blot membranes showed in Figure 2. a) Uncropped chemiluminescence and composite (chemiluminescence and brightfield) blots of Sdha of the CETSA showing Sdha‐1000 μM DMI interactions at 4 temperatures from +68 to +80°C; b) Uncropped chemiluminescence and composite (chemiluminescence and brightfield) blots of pAkt, Akt and β‐actin protein expression levels in control (vehicle‐treated) and 10 μM DMI‐treated NSCs after 24 hours.
**Figure S4:** Whole uncropped images of the original western blot membranes showed in Figure 5. a‐c) Uncropped chemiluminescence and composite (chemiluminescence and brightfield) blots of Sox2 (a), Cx26 (b), Cx45 (c) and the relative β‐actin (a‐c) protein expression levels in control (vehicle‐treated) and 10 μM DMI‐treated NSCs after 24 hours.
**Figure S5:** Whole uncropped images of the original western blot membranes showed in Figure 5. a‐c) Uncropped chemiluminescence and composite (chemiluminescence and brightfield) blots of Cx32 (a), Cx43 (b), Cx36 (c) and the relative β‐actin (a‐c) protein expression levels in control (vehicle‐treated) and 10 μM DMI‐treated NSCs after 24 hours.

## Data Availability

The data that support the findings of this study are available from the corresponding author upon reasonable request.
