# Supplementary material for: Connexin Regulation and Modulation of Neural Stem Cell Differentiation Induced by Cell‐Permeable Itaconate
Source: J Cell Physiol. 2026 May 11;241:e70185. doi: 10.1002/jcp.70185 (PMC13159055; doi:10.1002/jcp.70185)
Supplement: Supplementary file 1 — Figure S1: Characterisation of expression markers and proliferative dynamics in mouse SVZ‐derived NSCs. a) Representative confocal microscopy images of NSCs grown as monolayer (top panels) and neurospheres (bottom panels) expressing Sox2 (magenta) and Nestin (white). Nuclei were counterstained with Dapi (blue). b‐c) Linear growth curve and rate of cell growth (R.C.G.) of NSCs over 5 consecutive passages. Data are expressed as mean ± SEM of n = 8 independent replicates. Figure S2: a) Analysis of the absorbance at 450 nm of the medium+vehicle or the medium+1000 μM of DMI without cells. b) Cytofluorimetric analysis of 7‐AAD‐positive cells of NSCs cultures treated with 0, 0.1, 1, 10, 100 or 1000 μM of DMI at 24 hours. Data are shown as a % of gated cells and shown as dot plots and mean ± SEM of n = 4 independent replicates. Figure S3: Whole uncropped images of the original western blot membranes showed in Figure 2. a) Uncropped chemiluminescence and composite (chemiluminescence and brightfield) blots of Sdha of the CETSA showing Sdha‐1000 μM DMI interactions at 4 temperatures from +68 to +80°C; b) Uncropped chemiluminescence and composite (chemiluminescence and brightfield) blots of pAkt, Akt and β‐actin protein expression levels in control (vehicle‐treated) and 10 μM DMI‐treated NSCs after 24 hours. Figure S4: Whole uncropped images of the original western blot membranes showed in Figure 5. a‐c) Uncropped chemiluminescence and composite (chemiluminescence and brightfield) blots of Sox2 (a), Cx26 (b), Cx45 (c) and the relative β‐actin (a‐c) protein expression levels in control (vehicle‐treated) and 10 μM DMI‐treated NSCs after 24 hours. Figure S5: Whole uncropped images of the original western blot membranes showed in Figure 5. a‐c) Uncropped chemiluminescence and composite (chemiluminescence and brightfield) blots of Cx32 (a), Cx43 (b), Cx36 (c) and the relative β‐actin (a‐c) protein expression levels in control (vehicle‐treated) and 10 μM DMI‐treated NSCs after 24 hou [file JCP-241-0-s001.pdf]

Supplementary Material

Connexin Regulation and Modulation of Neural Stem Cell Differentiation Induced by Cell-permeable Itaconate

Simona Denaro[1], Simona Rosa Spina[1], Simona D'Aprile[2], Anna Gervasi[1], Filippo Torrisi[3], Carmela Parenti[3], Agata Zappalà[1,*], Nunzio Vicario[1,*], Rosalba Parenti[1].

[1] Department of Biomedical and Biotechnological Sciences, University of Catania, 95123 Catania, Italy.

[2] Department of Medicine and Surgery, University of Enna "Kore", 94100 Enna, Italy.

[3] Department of Drug and Health Sciences, University of Catania, 95123 Catania, Italy.

***Correspondence**: Agata Zappalà (email: azappala@unict.it; phone: +39 095 478 1324); Nunzio Vicario (email: nunziovicario@unict.it; phone: +39 095 478 1479).

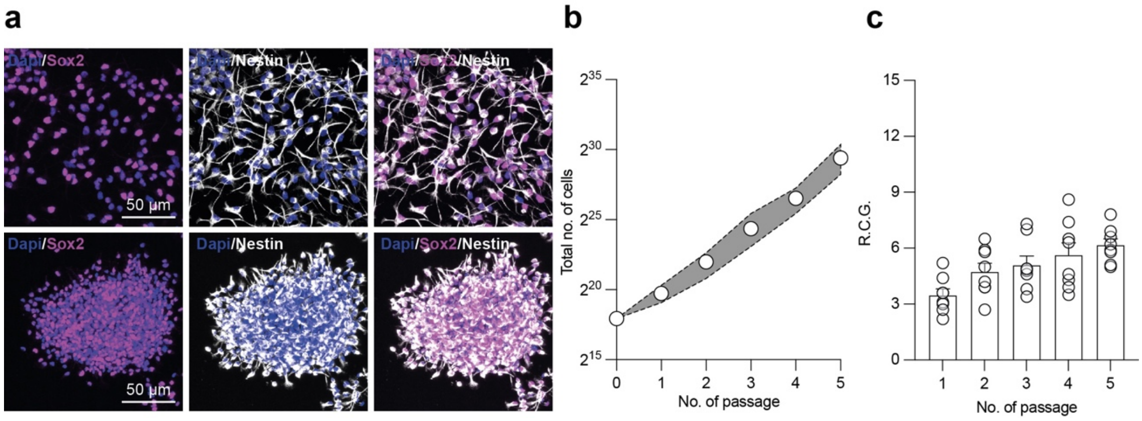

Figure S1. Characterisation of expression markers and proliferative dynamics in mouse SVZ-derived NSCs. a) Representative confocal microscopy images of NSCs grown as monolayer (top panels) and neurospheres (bottom panels) expressing Sox2 (magenta) and Nestin (white). Nuclei were counterstained with Dapi (blue). b-c) Linear growth curve and rate of cell growth (R.C.G.) of NSCs over 5 consecutive passages. Data are expressed as mean ± SEM of n = 8 independent replicates.

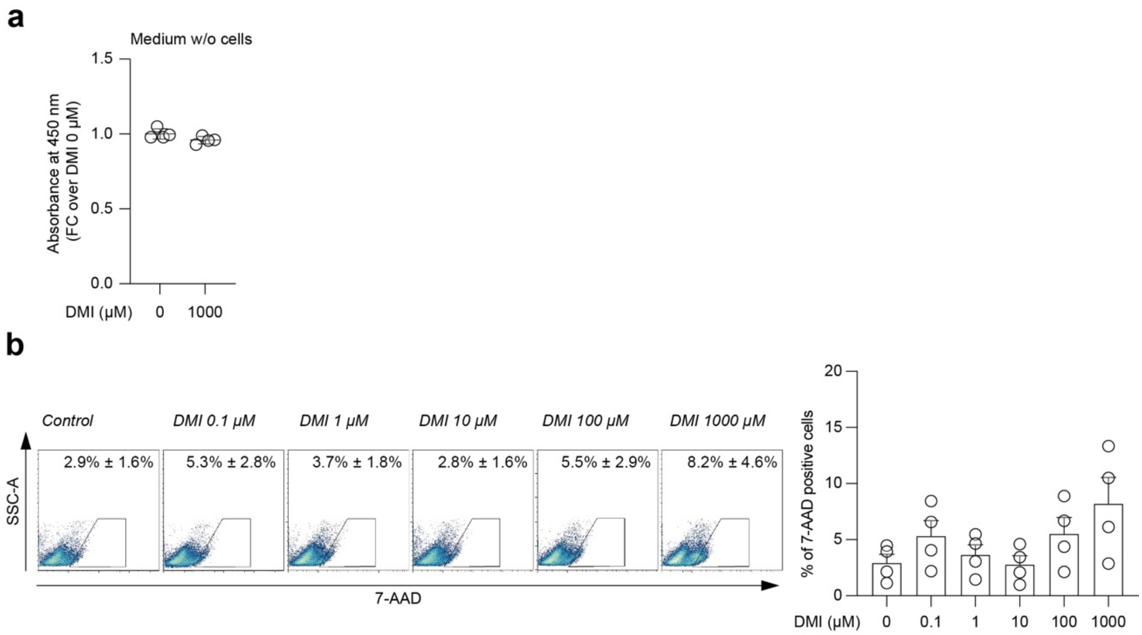

Figure S2. a) Analysis of the absorbance at 450 nm of the medium+vehicle or the medium+1000 µM of DMI without cells. b) Cytofluorimetric analysis of 7-AAD-positive cells of NSCs cultures treated with 0, 0.1, 1, 10, 100 or 1000 µM of DMI at 24 hours. Data are shown as a % of gated cells and shown as dot plots and mean ± SEM of n = 4 independent replicates.

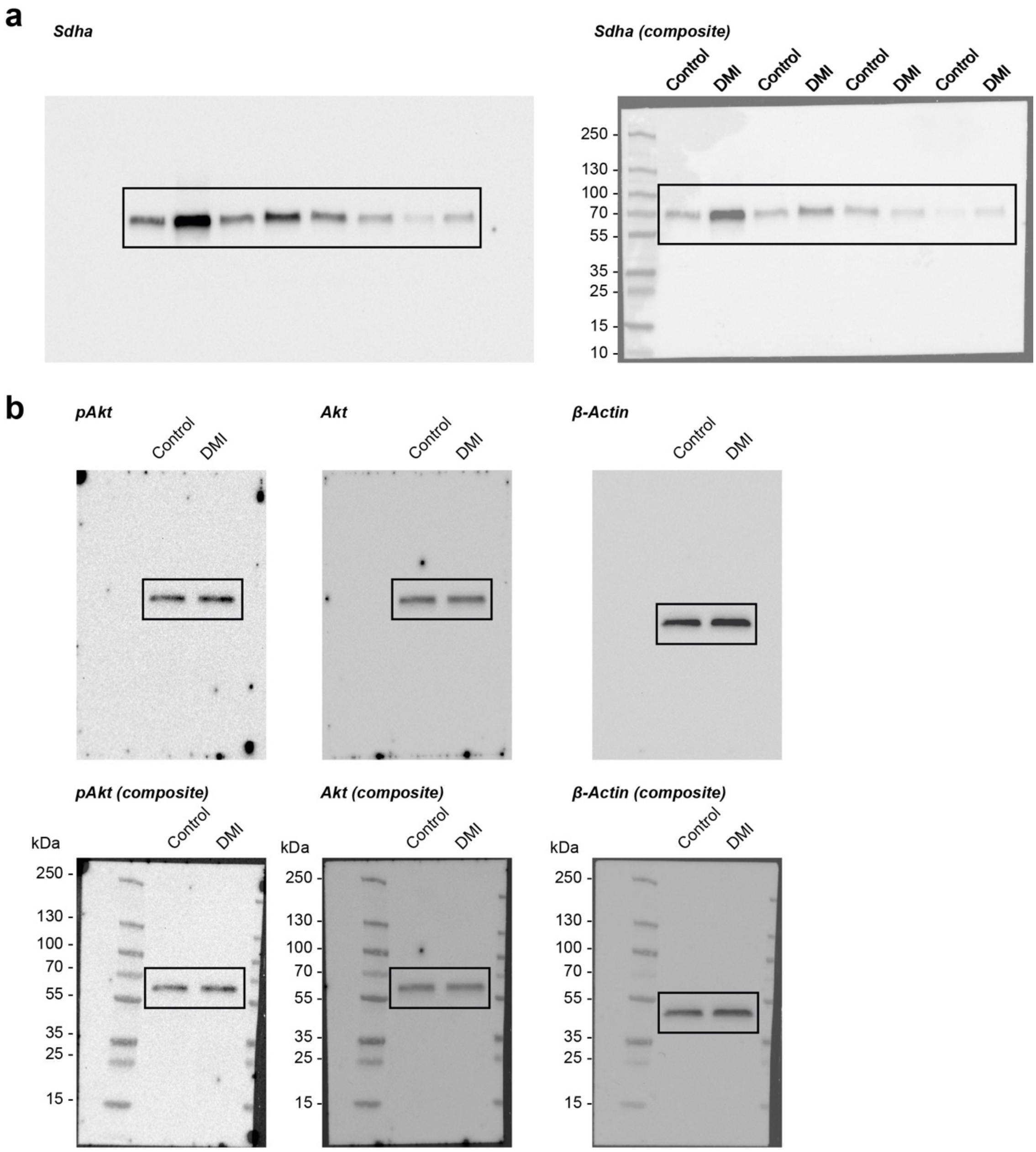

Figure S3. Whole uncropped images of the original western blot membranes showed in Figure 2. a) Uncropped chemiluminescence and composite (chemiluminescence and brightfield) blots of Sdha of the CETSA showing Sdha-1000 µM DMI interactions at 4 temperatures from +68 to +80 °C; b) Uncropped chemiluminescence and composite (chemiluminescence and brightfield) blots of pAkt, Akt and β-actin protein expression levels in control (vehicle-treated) and 10 µM DMI-treated NSCs after 24 hours.

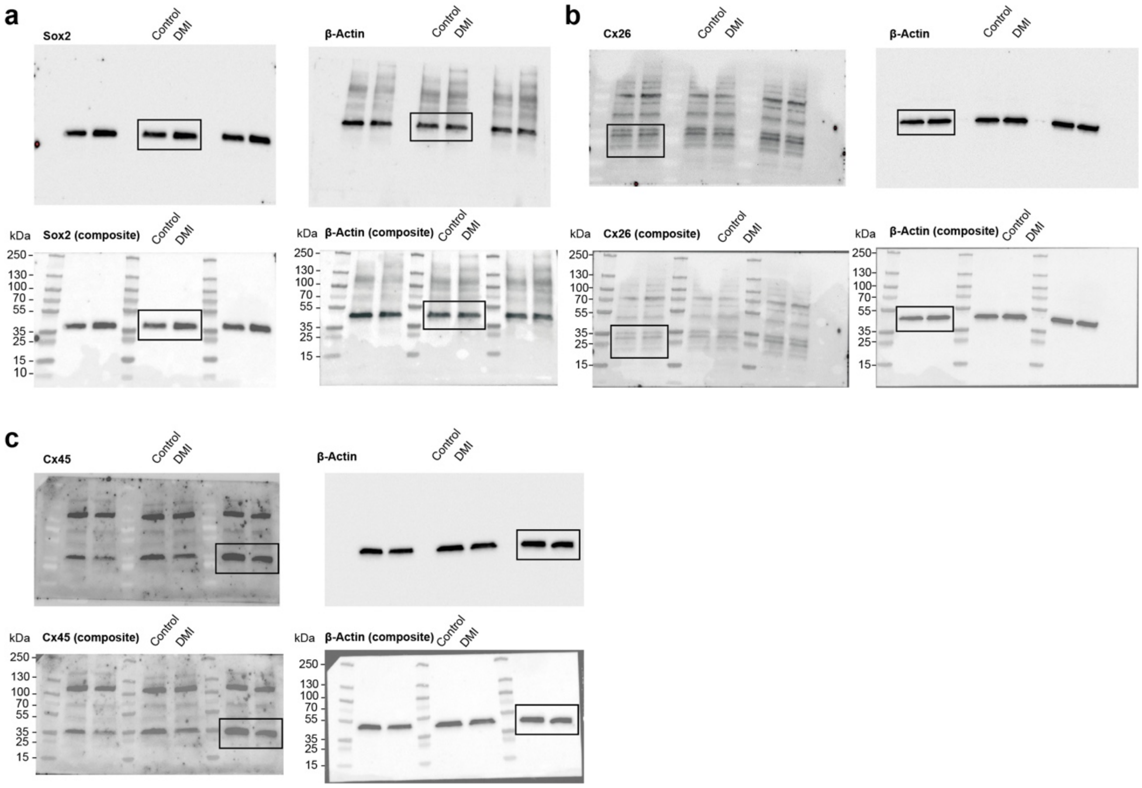

Figure S4. Whole uncropped images of the original western blot membranes showed in Figure 5. a-c) Uncropped chemiluminescence and composite (chemiluminescence and brightfield) blots of Sox2 (a), Cx26 (b), Cx45 (c) and the relative β-actin (a-c) protein expression levels in control (vehicle-treated) and 10 μM DMI-treated NSCs after 24 hours.

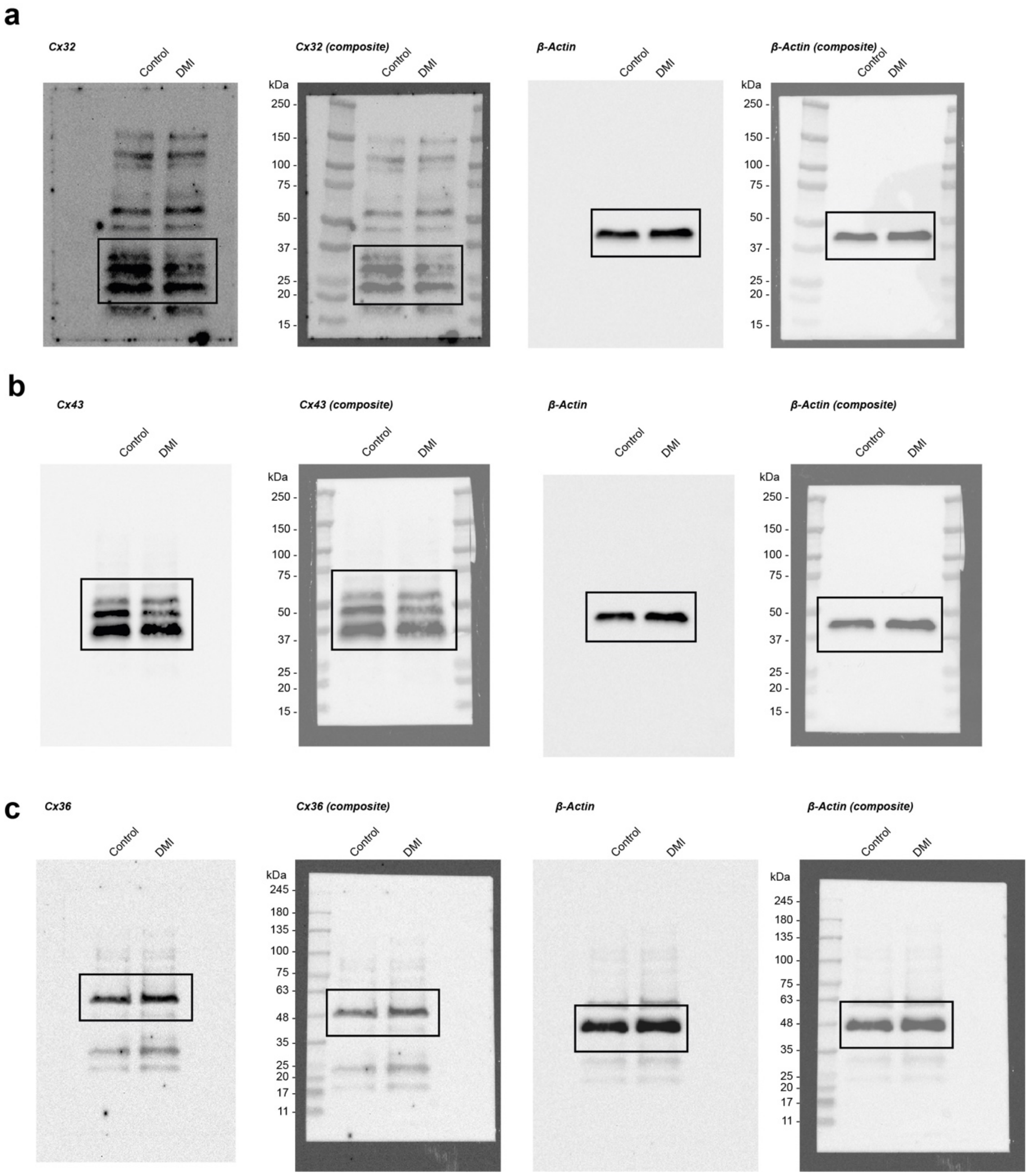

Figure S5. Whole uncropped images of the original western blot membranes showed in Figure 5. a-c) Uncropped chemiluminescence and composite (chemiluminescence and brightfield) blots of Cx32 (a), Cx43 (b), Cx36 (c) and the relative β-actin (a-c) protein expression levels in control (vehicle-treated) and 10 μM DMI-treated NSCs after 24 hours.
